# Downregulation of the NLRP3/Caspse-1 Pathway Ameliorates Ketamine-Induced Liver Injury and Inflammation in Developing Rats

**DOI:** 10.3390/molecules27092931

**Published:** 2022-05-04

**Authors:** Xinzhang Chen, Zhiheng Zhang, Meilun Shen, Xiangying Ma, Di Qiu, Siyao Li, Li Gao

**Affiliations:** 1 College of Veterinary Medicine, Northeast Agricultural University, Harbin 150030, China; xinzhangchen2022@163.com (X.C.); zzh449756020@163.com (Z.Z.); ml1604053343@163.com (M.S.); mxy0217212@163.com (X.M.); qd13836005047@163.com (D.Q.); dylisiyao@163.com (S.L.); 2 Heilongjiang Key Laboratory of Animals Disease Pathogenesis and Comparative Medicine, Harbin 150030, China

**Keywords:** ketamine, developing rats, livers, NLRP3/Caspase-1

## Abstract

Ketamine is an anesthetic drug that is widely used in human and veterinary medicine. In the developmental stage, long-term exposure to ketamine may cause serious side effects. MCC950 and VX765 play protective roles in many disease models by regulating the NLRP3/Caspase-1 pathway. This study aims to explore the potential protective effect of MCC950 and VX765 on ketamine-induced liver injury in neonatal rats and clarify its underlying mechanism. After administration of MCC950 and VX765 in a ketamine-induced liver injury rat model, liver function and inflammatory factors were determined, and immunohistochemistry and western blotting were performed. We found that ketamine caused liver injury in 7-day-old SD rats, decreased liver function indexes, and increased inflammation. MCC950 and VX765 effectively alleviated liver damage and inflammation, and downregulated the expression of proteins such as NLRP3, Caspase-1, and GSDMD-N. In summary, these results indicated that MCC950 and VX765 could have potential protective effects on ketamine-induced liver injury through inhibiting the NLRP3/Caspase-1 pathway.

## 1. Introduction

Ketamine is a *N*-methyl-d-aspartate (NMDA) receptor antagonist, which was identified in 1964 and mainly composed of (S)-ketamine and (R)-ketamine isomers. It is widely used as a preferred emergency and pediatric surgery sedation/analgesia agent in humans and in the veterinary clinic [1,2]. However, ketamine has a variety of side effects at anesthetic dosage [3,4]. Recent reports have presented adverse reactions related to ketamine, such as urotoxicity, hepatorenal toxicity, and memory impairment in humans [5]. In other studies, it has been shown that liver poisoning occurs occasionally after ketamine anesthesia, resulting in a significant increase in serum levels of transaminase [6,7], and liver injury has also been found in rabbits and mice [8,9]. These side effects in the liver rapidly disappear with the termination of anesthesia. As a commonly used anesthetic in clinical surgery, ketamine may also be exposed to anesthetics during the developmental stage of young animals. In previous studies, it has been shown that ketamine can cause nerve damage in 7-day-old rats [10]. However, the underlying mechanism of ketamine-induced liver injury remains unclear.

Furthermore, studies have shown that inflammatory responses in many liver diseases are caused by the release of IL-1β after the activation of inflammatory bodies in different liver cells [11]. Activation of the inflammasome induces the expression of inflammatory factors and recruits various immune cells to the damaged liver, which plays an important role in the cell pyroptosis pathway [12]. The typical pyroptosis pathway is triggered by various cytoplasmic sensor proteins [13]. NLRP3 and other sensing proteins can identify inflammatory factors and a variety of pathogens, recruit the pro-Caspase-1 monomer (pro-Caspase-1) through protein (ASC)-containing caspase recruitment domain (CARD) related to pyroptosis, and activate Caspase-1 through dimerization. Caspase-1 activation leads to Caspase-1 maturation and mediates inflammation [14], thereby regulating the cleavage and maturation of proinflammatory cytokines, such as IL-1β and IL-18 [15]. Increased levels of inflammatory cytokines (such as IL-1β and IL-18) induce pyroptosis in livers [16]. Therefore, regulating the pyroptosis pathway may be an effective way to alleviate ketamine-induced liver injury.

MCC950 (structural formula: C_20_H_24_N_2_O_5_S) is an NLRP3 inhibitor, which has been studied in a variety of inflammatory diseases caused by NLRP3 [17,18]. In the latest research report, it was believed that MCC950 directly interacted with the Walker B motif in the NLRP3-NACHT domain, thereby blocking ATP hydrolysis, inhibiting NLRP3 activation, and inflammatory corpuscle formation [19]. VX-765 (structural formula: C_24_H_33_ClN_4_O_6_), a Caspase-1 inhibitor, inhibits Caspase-1 activation and cleavage of precursors IL-1β and IL-18 [20]. However, whether these two inhibitors can protect ketamine-induced pyroptosis of liver cells remains to be elucidated. In this study, we investigated the role of the pyroptosis pathway in ketamine-induced liver injury in 7-day-old rats. Our findings showed that MCC950 and VX765 inhibitors could alleviate the degree of pyroptosis of liver cells through the NLRP3/Caspase-1 pathway, thereby alleviating ketamine-induced liver injury in developing rats. Taken together, these results provide a reference for the clinical medication of developing animals, reducing the impact of anesthetics on developing animals, and providing novel therapeutic targets.

## 2. Results

### 2.1. Effects of MCC950 and VX765 Ketamine on Liver Function in Rats

**Figure 1:**
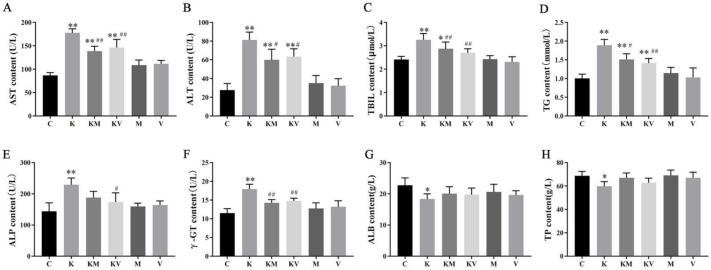
Effects of MCC950 and VX765 on ketamine induces liver function injury in neonatal rats. Serum level of (**A**) AST, (**B**) ALT, (**C**) TBIL, (**D**) TG, (**E**) ALP, (**F**) γ-GT, (**G**) ALB, and (**H**) TP (*n* = 6). Results are the mean ± SD. * *p* < 0.05 vs. control group, ** *p* < 0.01 vs. control group; # *p* < 0.05 vs. ketamine group, *p* < 0.05; ## *p* < 0.01 vs. ketamine group.

To explore the effect of MCC950 and VX765 on liver function, we detected the levels of liver biochemical indices. As shown in Figure 1, we found that after treatment with ketamine, the levels of ALT, AST, TBIL, TG, ALP, and γ-GT in group K significantly increased; however, the levels of ALB and TP in group K decreased when compared with group C. The levels of ALT, AST, TBIL, TG, ALP, and γ-GT were markedly lower in the KM group as compared with K group, while the levels of ALB and TP showed no significant changes. There were no obvious changes when treated with MCC950 or VX765 on normal rats (Figure 1A–H). Taken together, these findings indicated that MCC950 and VX765 can reduce ketamine-induced liver damage.

### 2.2. Effects of MCC950 and VX765 Ketamine on Histopathological Changes in Rats

**Figure 2:**
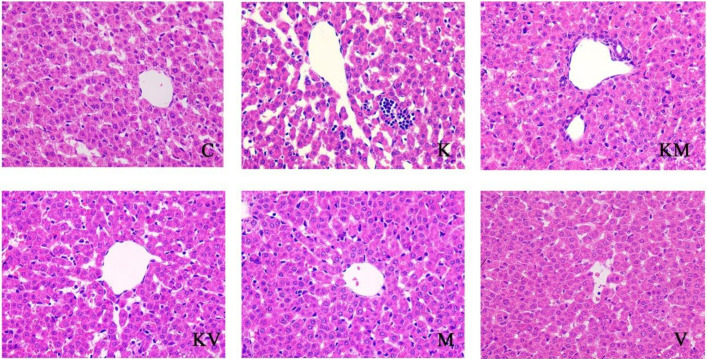
Liver sections for histological examination by hematoxylin and eosin staining (H&E) at ×400 (*n* = 3).

Furthermore, the histological changes in the liver were observed using H&E staining. The results showed that the boundary of liver tissue was clear and complete, and the arrangement of liver cells was normal in the control group (Figure 2). However, obvious hepatic sinusoid dilatation, extensive inflammatory infiltration of hepatocytes, an unclear boundary of the liver tissue, cell swelling, and scattered vacuolar degeneration were observed in the ketamine group. In contrast, the swelling of liver cells was alleviated, and the liver cells were still arranged in a cord shape, showing a minor infiltration of inflammatory cells in the KM and KV group, indicating MCC950 and VX765 significantly alleviated the damage of liver tissue as compared with the ketamine group. In the single inhibitor group (M group and V group), the morphology of liver tissue was relatively normal, and no obvious pathological changes were observed.

### 2.3. Effects of MCC950 and VX765 on Inflammatory Cytokines in Rats

**Figure 3:**
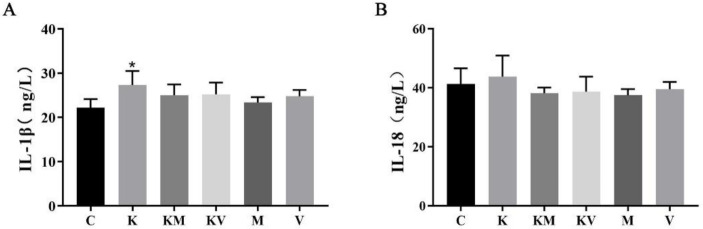
Effects of MCC950 and VX765 on inflammatory cytokines in neonatal rats. (**A**) IL-1β; (**B**) IL-18 (*n* = 6). Results are the mean ± SD. * *p* < 0.05 vs. control group.

To further detect the levels of inflammatory cytokines, we performed ELISA to examine the levels of IL-1β and IL-18 in livers. As shown in Figure 3, the levels of IL-1β and IL-18 in the ketamine group were increased when compared with the control group. After treatment with inhibitors, the KM and KV groups showed a decreasing trend compared with rats in the ketamine group.

### 2.4. Effects of MCC950 and VX765 on NLRP3/Caspase-1 Pathway in Rats

**Figure 4:**
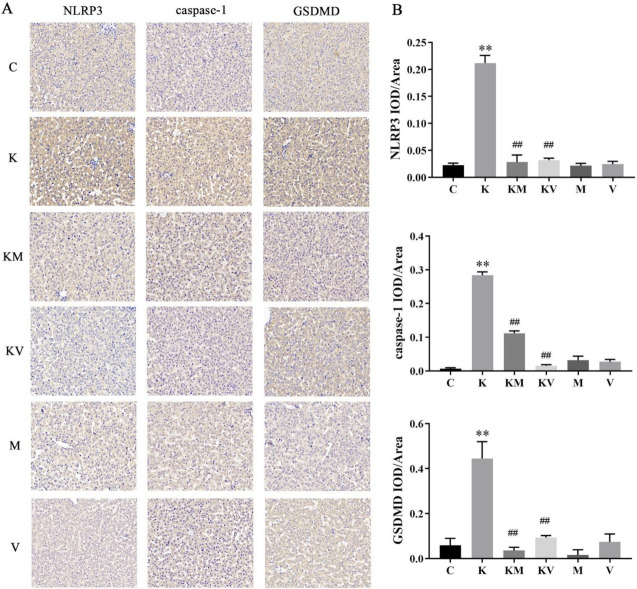
Immunohistochemical staining and analysis. (**A**) Immunohistochemical representative images of NLRP3-3, Caspase-1, and GSDMD in liver at ×400. (**B**) Immunohistochemical analysis for mean density (*n* = 3). Results are the mean ± SD. ** *p* < 0.01 vs. control group; ## *p* < 0.01 vs. ketamine group.

**Figure 5:**
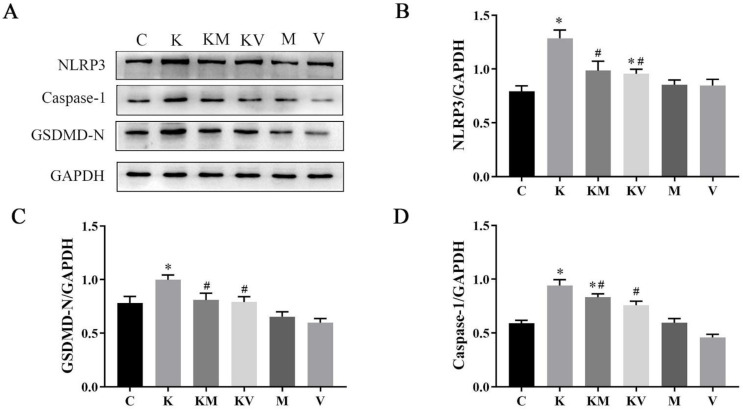
Effects of MCC950 and VX765 on ketamine induces pyroptosis in liver of neonatal rats. (**A**) Western blotting bands and (**B**, **C** and **D**) representative image for NLRP3-3, Caspase-1 p20, and GSDMD-N (*n* = 3). Results are the mean ± SD. * *p* < 0.05 vs. control group; # *p* < 0.05 vs. ketamine group.

To confirm whether MCC950 and VX765 play an important role in liver protection by interfering with the NLRP3/Caspase-1 signaling pathway, NLRP3, Caspase-1, and GSDMD were determined by immunohistochemistry (Figure 4) and Western blot analysis (Figure 5). The results showed that the expression of NLRP3, Caspase-1, and GSDMD was significantly increased after ketamine administration. Compared with the ketamine group, MCC950 and VX765 inhibitors significantly decreased these proteins’ expression, thereby indicating that ketamine effectively inhibited activation of the NLRP3/Caspase-1 pathway.

## 3. Discussion

In our study, we demonstrated that MCC950 and VX765 reduced ketamine-induced injury and inflammation in the liver of developing rats by inhibiting the activation of the NLRP3/Caspase-1 pathway, thereby protecting the liver of developing rats. Taken together, MCC950 and VX-765 may be potential protective compounds that reduce liver injury caused by continuous exposure to ketamine.

Evidence showed that low-dose ketamine treatment reduced glycogen levels in the liver of rats, and they showed sustained behavioral changes and weight loss, which may be due to the failure of liver glycogen synthesis or increased glycogenolysis [21]. After using a high dose ketamine for two weeks, pathological changes in the liver and mitochondrial degeneration were observed in rats [22,23,24]. In other studies, ketamine was found to promote the degradation of the biological liver function, resulting in gluconeogenesis [25], increasing glycogenolysis [26], and elevated blood glucose levels [27,28]. In a chronic liver injury model, liver function indexes, such as ALT, AST and TBIL were significantly increased, and H&E and oil red O staining also showed that liver injury occurred in mice [29]. In our study, we proved that compared with the control group, liver function indexes after ketamine anesthesia were significantly upregulated, and albumin and total protein levels showed a downward trend. In pathological tissue sections, we also found that ketamine caused obvious pathological changes in liver tissue, indicating that ketamine can cause liver damage in young rats. These findings suggested that a liver injury model was successfully established after continuous exposure to ketamine for a short time. 

Qu et al. found that MCC950 can significantly reduce cholestatic liver injury by reducing the production of proinflammatory cytokines IL-1β and IL-18 and by inhibiting neutrophil infiltration and hepatocyte death [30]. In a nonalcoholic fatty liver disease (NAFLD) model, MCC950 improved the pathological state of NAFLD and liver fibrosis in obese diabetic mice [31]. These results showed that MCC950 can play a protective role in liver injury. VX765 could penetrate the blood–brain barrier and is rapidly metabolized to VRT-043198 in vivo, thereby targeting Caspase-1 [20]. Several studies have confirmed that VX765 has a protective effect on the nervous system. VX765 reduced the expression of inflammatory corpuscles and pyroptosis-related proteins in the central nervous system and improved neural behavior in autoimmune encephalomyelitis animals [17]. Moreover, in a mouse model of sepsis, VX765 played a brain protective role in subcortical arteriosclerotic encephalopathy and cognitive impairment [32]. In our study, we observed that liver function was restored as compared with the ketamine group; liver function indicators showed a clear trend of recovery after intervention with MCC950 and VX765. These two inhibitors reduced the infiltration of inflammatory cells and cell swelling in the liver; the overall morphology of liver tissue was better. These findings indicated that ketamine could alleviate ketamine-induced liver damage in young rats.

In the uninfected liver, both metabolism and tissue remodeling require inflammatory components. After immune activation to resist pathogens or tissue damage, solving inflammation and other stimuli is essential for maintaining liver homeostasis. Inflammatory responses should immediately and effectively controlled and existing stimulus factors should be eliminated in time to avoid excessive inflammatory responses [33]. Treatment with MCC950 decreased the serum levels of IL-1β and IL-6, ALT/AST, and the severity of liver inflammation [31]. In our study, treatment with MCC950 and VX765 showed an effective reversal of liver inflammation.

Various studies have shown that the production of inflammatory factors is mediated by the pyroptosis pathway. Pyroptosis can cause channel-mediated cell dissolution and IL-1β secretion [34]. In the pyroptosis pathway, several dangerous signals can activate the cytoplasmic innate immune signal receptor NLRP3. The activation of NLRP3 makes the assembly and nucleation of inflammatory cells possible, which leads to the cleavage of GSDMD, thus causing cell pyroptosis and inflammation [35]. In previous studies, it has been shown that a new mouse model can lead to excessive activation of NLRP3 inflammatory bodies in hepatocytes, thereby shortening the survival period of mice and causing poor growth and severe liver inflammation. This shows that excessive activation of inflammation leads to the pyroptosis of mouse liver cells [36]. Chu et al. found that patulin-induced pyroptosis in mouse liver cells depended on activation of the NLRP3 inflammasome. After treatment of the cells with NLRP3 inhibitor MCC950, the expression levels of Caspase-1 and IL-1β were significantly decreased [37]. VX-765 can also effectively inhibit the activation of Caspase-1 in asthmatic mice and reduce the levels of IL-18 and IL-1β, accompanied by a significant decrease in airway inflammation [38]. In other studies, it was shown that MCC950 could effectively alleviate diabetes-induced renal damage by inhibiting the NLRP3/Caspase-1 pathway, which may be a potential therapeutic strategy for preventing the progression of diabetic nephropathy [39]. These results showed that MCC950 and VX765 inhibitors could effectively improve different disease models. In our study, MCC950 and VX765 treatment effectively downregulated the levels of NLRP3, Caspase-1, GSDMD-N and other indicators, and the same trend was also verified in the immunohistochemistry. These findings indicated that MCC950 and VX765 could inhibit the activation of inflammatory bodies in the liver, thereby alleviating ketamine-induced pyroptosis of liver cells in young rats. Therefore, we speculate that inhibiting the activation of inflammasomes and blocking the activation of downstream Caspase-1 is one of the potential mechanisms to alleviate the liver injury caused by ketamine. MCC950 and VX765 may have a protective effect on ketamine-induced liver injury in young rats by regulating the NLRP3/Caspase-1 pathway.

## 4. Materials and Methods

### 4.1. Animals and Drugs Treatment

Seven-day-old Sprague Dawley (SD) rats (20 g ± 2 g) were housed on a standardized pelleted diet and supplied with tap water. The rats were purchased from the Animal Center of Harbin Medical University (Harbin, China). All experimental procedures and protocols were carried out in accordance with the guidelines of the Experimental Animal Ethics Committee of Northeast Agricultural University.

The 7-day-old SD rats were randomly divided into 6 groups: the control group (group C), the ketamine group (group K), the ketamine + MCC950 group (group KM), MCC950 group (group M), the ketamine + VX765 group (group KV), and the VX765 group (group V). For ketamine treatment, 7-day-old rats received 20 mg/kg ketamine (Zhongmu Beikang Pharmaceutical Co., Ltd. Jiangsu, China) intraperitoneally every 90 min five times. For drug treatment, 10 mg/kg MCC950 (MedChemExpress, MCE, Shanghai, China; HY-12815) or 25 mg/kg VX765 (MCE, HY-13205) was administered intraperitoneally to the rats 30 min before the ketamine injections. The control group, group KV) and group V were given a normal, 10 mg/kg MCC950 or 25 mg/kg VX765, respectively. At the end of 90 min after the last dose of ketamine, the rats were euthanized, and liver tissue and serum were collected for the following experiments.

### 4.2. Liver Function Assay

The contents of aspartate aminotransferase (AST), alanine aminotransferase (ALT), alkaline phosphatase (ALP), γ-glutamyl transpeptidase (γ-GT), total protein (TP), albumin (ALB), total bilirubin (T-BIL), and triglyceride (TG) were performed according to the manufacturers’ recommendations at the Nanjing Jiancheng Bioengineering Institute (Nanjing, China).

### 4.3. Histopathological Examination

After a fixative and dehydrating process, the liver tissues were placed in a paraffin-embedded box. The embedded tissue was then cut into 5 μm sections. Sections were baked in a drift temperature controller for 2 h, removed, placed at room temperature, and then saved in a section box. Paraffin sections were soaked, dewaxed, then washed with distilled water. Sections were stained with hematoxylin and eosin (H&E) and then washed with tap water. After the sections were dried, they were sealed with neutral gum. The pathological changes in the liver were observed under a microscope.

### 4.4. Enzyme-Linked Immunosorbent Assay (ELISA)

The contents of IL-1β and IL-18 in the livers were measured using ELISA kits (Jingmei Biological Technology Co., Ltd., Jiangsu, China) according to the manufacturer’s instructions.

### 4.5. Immunohistochemistry (IHC) Staining

After deparaffinization, H_2_O_2_ was added, and the sections were incubated for 10 min; then blocked with goat serum at 37 °C for 1 h. The sections were then incubated with anti-NLRP3 rabbit polyclonal antibody (1:200; ABclonal, A12694), anti-caspase-1 rabbit polyclonal antibody (1:150; ABclonal, A0964), or anti-GSDMD rabbit polyclonal antibody (1:200; Affinity, AF4012) at 4 °C overnight. Horseradish peroxidase-(HRP-) conjugated secondary antibody was applied and stained with a diaminobenzidine (DAB) kit (Servicebio, Wuhan, China). Image-Pro Plus software 6.0 (Media Cybernetics, Rockville, MD, USA) was used to analyze the integral optical density (IOD) value of integral optical density from 3 different regions.

### 4.6. Western Blotting Analysis

Total protein quantification was performed using the BCA protein assay kit (Beyotime, Shang Hai, China), and lysed protein was separated by 10–12% SDS-PAGE gel and transferred to a nitrocellulose membrane. Protein levels were determined via incubation with antibodies against NLRP3 (ABclonal, A14223, 1:1000), caspase-1 (ABclonal, A0964, 1:1000), GSDMD (ABclonal, A10164, 1:1000), and GAPDH (ABclonal, A19056, 1:1000). The blots were detected with an ECL kit (Beyotime, Shanghai, China), imaged using a Gel Imaging System (Tanon5200, Tanon, China). Image J software (Version 1.8, USA) was used to determine the densities of the protein bands.

### 4.7. Statistical Analysis

All statistical analyses were performed using SPSS software (Version 22.0, USA). One-way ANOVA was performed for comparisons between groups, and multiple comparisons between groups were performed using a post hoc Tukey test. The results were expressed as the mean ± standard deviation (Mean ± SD). *p* < 0.05 was considered statistically significant.

## 5. Conclusions

In this study, treatment with MCC950 and VX765 improved ketamine-induced liver injury in 7-day-old rats and effectively inhibited activation of the NLRP3/Caspase-1 pathway. This study might be provided a underlying therapeutic scheme for the treatment of ketamine-induced liver injury in young rats. 

## Data Availability

The data presented in this study are available in the manuscript.
